# Infodemic and the fear of monkeypox: call for action

**DOI:** 10.1186/s41182-022-00459-8

**Published:** 2022-09-05

**Authors:** Ramadan Abdelmoez Farahat, Michael G. Head, Samar Tharwat, Yasmeen Alabdallat, Mohammad Yasir Essar, Basel Abdelazeem, Mounir Ould Setti

**Affiliations:** 1 Faculty of Medicine, Kafrelsheikh University, Kafrelsheikh, 33511 Egypt; 2 Global Research Group (GRG), Kafrelsheikh, Egypt; 3 Clinical Informatics Research Unit, Faculty of Medicine, University of Southampton, Southampton, UK; 4 Rheumatology and Immunology Unit, Internal Medicine Department, Faculty of Medicine, Mansoura University, Mansoura, Egypt; 5 Faculty of Medicine, Hashemite University, Zarqa, Jordan; 6 Kabul University of Medical Sciences, Kabul, Afghanistan; 7 Department of Internal Medicine, McLaren Health Care, Flint, MI 48532 USA; 8 Department of Internal Medicine, Michigan State University, East Lansing, MI 48823 USA; 9 Institute of Public Health and Clinical Nutrition, University of Eastern Finland, Kuopio, Finland; 10 Global Database Studies, IQVIA, Espoo, Finland

**Keywords:** Monkeypox, Social media, Infodemic, Stigma, Fake news

## Abstract

Monkeypox (MPX) was declared a public health emergency of international concern by the World Health Organization (WHO), as of July 23^rd^, 2022. Fake news spread on social media has already surfaced and contributed to worsening of this concerning situation, making it difficult for the health care experts’ voices to be heard. Therefore, we recommend some solutions to overcome this situation, including raising public awareness and preventing stigma through sharing engagement with civil society organizations, and better cooperation between policymakers, the medical community, and social media platforms regarding providing accurate official news about MPX. WHO-one health approach should be established and prioritized.

Monkeypox (MPX) has infected more than 40,932 cases across 90 countries as of August 22nd, 2022 [1]. MPX was declared a public health emergency of international concern by the World Health Organization (WHO), as of July 23rd, 2022. Although MPX is transmitted through close contact with infected lesions, respiratory droplets, bodily fluids, and contaminated objects (i.e., fomites), it has mainly spread among males who have sex with males [2]. There has been extensive coverage of the outbreak from mainstream news agencies and social media sites.

Fake news is defined as the dissemination of incorrect information using digital or traditional media. Users' newsfeeds on social media sites (i.e., Facebook) are typically filled with their likes and beliefs. This creates an environment where others with similar beliefs can distribute false information to one another, making it difficult for the expert commentary of healthcare professionals or public health services to be heard [3].

When confronted with something utterly uncharted, especially due to misinformation spread on social media, people are sometimes wary of governments [4]. During the COVID-19 pandemic, many fake pages and even well-known public figures contributed negatively to the crisis by spreading fake news, putting the public's trust in their governments to the test [5]. Vaccine hesitancy was also enhanced due to public mistrust of public and governmental authorities [5].

"Infodemic" refers to the impact of widespread dissemination of disease-related information, including false or misleading information, as reported by WHO [3]. Massive infodemics have accompanied the COVID-19 pandemic. The WHO resources include guidance around the art of ‘story-telling’. Misinformation narratives that go viral can be countered by story-telling with positive messages and here, public trusted figures (perhaps including celebrities or other famous individuals) can provide a positive input [6].

Untruths and misconceptions about the MPX, which are not well known, have already surfaced on social media and other platforms. These false claims include those repeated during the COVID-19 pandemic, for example, that MPX was created in a laboratory or that there is a link between COVID-19 vaccines and MPX prevalence [7]. This can also create stigma, particularly so here against gay communities who are broadly considered to be at the highest risk of monkeypox infection. High-level global groups such as UNAIDS have released statements urging “a rights-based, evidence-based approach that avoids stigma” [8]. The WHO is also considering renaming monkeypox, due to concerns around racism and media reporting that used photos of MPX lesions on Afro-Caribbean skin despite the outbreaks being mainly in countries with predominantly Caucasian populations [9].

Stigma can be addressed using mitigation frameworks [10] specifically modified for monkeypox that allow consideration of factors such as individual and family dynamics. This can then be used by civil society organizations to listen and co-create pertinent, useful, and accurate information for the general public, priority groups and communities It is vital that potential cases or those potentially exposed to MPX come forward to their local health services, and these individuals must be confident they will receive good discrete care and be given good public health advice.

To conclude, we recommend cooperation between policymakers, healthcare providers, mainstream media, and social media platforms by providing accurate official news about MPX, debunking circulating myths and misinformation. Mitigation frameworks can support consideration of multiple factors to proactively reduce potential sources of stigma. Public figures (for example, senior politicians or national leaders, social media activists, and famous celebrities) and the medical community can play a vital role by providing reassurance and communicating public health guidelines. There are numerous research gaps where the evidence base is weak or non-existent and expedited studies can be aiming to investigate most appropriate public health messaging and best practice in locally tailored health promotion. Policymakers can also review modes of communication between the healthcare worker and patient to build trust and confidence, thus promoting active and early seeking of healthcare.

## Data Availability

None.

## Abbreviations

MPX: Monkeypox
MPXV: Monkeypox virus
WHO: World Health Organization
